# Madagascar 2018-2019 measles outbreak response: main strategic areas

**DOI:** 10.11604/pamj.2020.37.20.24530

**Published:** 2020-09-05

**Authors:** Vincent Dossou Sodjinou, Alfred Douba, Marcellin Mengouo Nimpa, Yolande Vuo Masembe, Mireille Randria, Charlotte Faty Ndiaye

**Affiliations:** 1 World Health Organization Regional Office for Africa, Congo,; 2 World Health Organization Country Office, Madagascar,; 3 Felix Houphouet Boigny University, Abidjan, Côte d´Ivoire

**Keywords:** Measles, outbreak response, Madagascar

## Abstract

**Introduction:**

on October 4^th^, 2018, a measles outbreak was declared in Madagascar. This study describes the outbreak response in terms of coordination, case management, vaccination response and epidemiological surveillance.

**Methods:**

data were collected using a line list and vaccination tally sheet. Serum samples were collected within 30 days of rash onset for laboratory testing; confirmation was made by detection of measles immunoglobulin M antibody.

**Results:**

from September 2018 to May 2019, a total of 146,277 measles cases were reported which included 1394 (1%) laboratory-confirmed cases and 144,883 (99%) epidemiological link-confirmed cases. The outbreak affected equally males (72,917 cases; 49.85%) and females (73,233 cases; 50.06%). The sex was not specified for 127 (0.09%) cases. Case fatality rate and attack rate were high among children less than 5 years. Responses interventions include effective coordination, free of charge case management, reactive vaccination, strengthened real-time surveillance, communication and community engagement and the revitalization of the routine immunization. Reactive vaccination was implemented in different phases. A total of 7,265,990 children aged from 6 months to 9 years were vaccinated. Post campaign survey coverage was 95%, 96% and 97% for phase 1, 2, 3 respectively.

**Conclusion:**

elimination of measles will be challenging in Madagascar because of low routine immunization coverage and the absence of a second dose of measles vaccine in the routine immunization schedule.

## Introduction

Measles is one of the most infectious human diseases which can cause serious illness, lifelong complications and death. Globally, an estimated 535,000 children died of measles in 2000. Most of these deaths occurred in developing countries and measles accounted for 5% of all under five mortality. Efforts for measles control led to a 74% global reduction of measles deaths between 2000 and 2010 [1]. In 2011, the World Health Organization (WHO) African region adopted a strategy and a resolution for measles elimination in the region by 2020. The targets adopted for 2020 are: measles incidence of less than 1 case per million population; maintaining 95% measles immunization coverage at national level and in all districts; attaining 95% coverage in all scheduled measles supplementary immunization activities (SIAs) and in response to outbreaks; and maintaining the targets for the two main surveillance performance indicators [2]. These indicators include an annual non measles febrile rash illness rate of at least 2 per 100,000 population and annual proportion of at least 80% of districts that have reported at least 1 suspected case of measles with a blood specimen [3]. From 2007 to 2016, 4 measles supplemental immunization activities (SIAs) were implemented in Madagascar leading to a decrease of measles incidence from 330 cases per 1,000,000 inhabitants in 2000 to 0.2 case per 1,000,000 inhabitants in 2016. In addition, measles administrative coverage was above 80% from 2014 to 2018 and surveillance activities were conducted [4]. However, in 2017, African countries evaluation towards measles elimination by 2020 showed that Madagascar was among countries significantly off-track for achieving the elimination goal [5]. In addition, in 2016, of 114 health districts in the country, 86 (75%) achieved an administrative coverage of at least 95% [6]. Eventually, on October 4^th^, 2018, a measles outbreak was confirmed by the national reference laboratory. This outbreak started in the capital city, Antananarivo and extended to all the 22 regions of Madagascar [7,8]. In response to the outbreak, several interventions were conducted. In this article we describe coordination, case management, vaccination response and epidemiological surveillance during the outbreak response.

## Methods

**Setting:** Madagascar is the fourth biggest island country in the world. Located in the Indian Ocean, it covers 587041km^2^ and is separated from the African continent by the Mozambique channel. The country is divided in to 22 administrative regions and 114 health districts. In 2018, the total population was estimated at 26,330,637 inhabitants (49.9% males). Twenty percent (20%) of the population live in urban area [4].

**Data collection:** data were collected using minutes of coordination committee meetings, activities reports, line list and vaccination tally sheet. A line list was developed for the outbreak. Line list was available in health facilities. Patient´s information was recorded on the line list and a blood specimen is collected when he/she visited the health facility. Active search of cases was conducted by community workers for patient who did not visit the health facility. Information of patients were sent to the health facility and patients were asked to visit the health facility for management. Variables in the line list included the name of health district, year, suspected disease, epidemiological week, patient´s name, health facility´s name, residence place´s name, sex, age, date of rash onset, health facility visit date, symptoms (maculopapular eruption, fever, conjunctivitis, cough, coryza), immunization status (vaccinated against measles, date of vaccination, not vaccinated), lab test (blood sample, date of sampling, lab result), outcome (alive, dead, unknown), places visited 2 weeks prior the beginning of the illness and comment (uncommon sign, hospital, community). A tally sheet was developed for the vaccination campaign. The tally sheet had 2 sections: one section for children aged less than 1 year and the other section for children aged 1 to 9 years. Vaccination teams utilized the tally sheet for the recording of children vaccinated. In each district, tally sheets were sent to vaccination focal point by all vaccination teams on a daily basis. Vaccination data were compiled by the focal point and sent to the expanded programme on immunization (EPI) every day. Data from case investigations form and line listing were entered in Epi-info in standard measles surveillance database. Those of vaccination campaigns were entered into Epi-info Microsoft Excel for analysis. Population estimates obtained from the 1993 census were adjusted for 2.8% population growth rate [9] and used for calculating measles incidence and vaccination coverage.

**Case definition and laboratory test:** a suspected measles case was defined as: any person with generalized maculopapular rash and fever plus one of the following: cough or coryza (runny nose) or conjunctivitis (red eyes); any person in whom a clinician suspects measles. Measles suspected cases at community level was defined as any person with generalized rash and fever. For each suspect case, prior to outbreak confirmation, serum samples were collected within 30 days of rash onset for laboratory testing; confirmation was made by detection of measles immunoglobulin M (IgM) antibody at an accredited national measles laboratory using a standard commercial enzyme immunoassay indirect kit [10]. Laboratory confirmed measles was defined as a suspected measles case that is investigated, including the collection of blood specimen, has serological confirmation of recent measles virus infection (measles IgM positive) and had not received measles vaccination in the 30 days preceding the specimen collection. Measles confirmed by epidemiological linkage was defined as a suspected measles case that has not had a specimen taken for serologic confirmation and is linked (in place, person and time) to lab confirmed cases; i.e. living in the same or in an adjacent district with a lab confirmed case where there is a likelihood of transmission; onset of rash of the two cases being within 30 days of each other. A confirmed outbreak of measles was defined as 3 or more measles IgM positive (laboratory confirmed) cases in a health facility or district in one month [3].

**Outbreak management:** outbreak confirmation was followed by a rapid grading of the outbreak within 72 hours by the WHO which classified it as a grade 2 of emergency response framework, meaning that country need support to cope with this outbreak. Therefore, outbreak management by the ministry of health and partners was based on the WHO Incidence management system. The response plan covered main strategic areas: coordination with ministry of health and partners; case management; supplementary immunization mass campaign with measles vaccines; laboratory and epidemiological system; communication; logistic and supplies.

**Coordination and resources mobilisation:** the government and partners set up a three level coordination mechanism: a strategic coordination committee, a technical coordination committee and multisectoral coordination committee. The first two task forces, under the leadership of the ministry of health included the ministry of health and all the partners including the private sector, had weekly meeting. The multisectoral committee was under the leadership of the prime minister and included different departments and bilateral and multilateral partners. Beside theses task forces, WHO established weekly partners´ coordination meeting to ensure coordinated support to the government and complementarity of partners´ interventions.

**Case management:** standard treatment protocols were developed based on the WHO recommendations. Treatment was deliver free of charge. All cases received vitamin A in age appropriate doses and were hospitalized for treatment of complications.

**Supplementary measles mass vaccination campaigns:** three round of measles mass campaigns were organized respectively in January, March and April 2019. The first round targeted children 9 months - 9 years while the two others were extended to children 6 months - 9 months. Extension below 9 months was based on the fact that many cases were registered among young children with high case fatality death. For each vaccination round, microplanning was organized and health care workers were trained using official training material. A variety of strategies were used including fixed and temporary posts and schools to schools strategy. Very late in the outbreak, immunoglobulin (IMIG) became available for contacts of cases with contraindications to vaccination including infants aged less than 6 months, pregnant women and immunocompromised persons.

**Strengthening epidemiological surveillance and laboratory capacities:** rapid investigation of the outbreak in 72 hours following confirmation enable better understanding of the outbreak cause: non-vaccination of children and the most appropriate age group to be targeted by immunization campaigns. Health personnel were sensitized on how to recognize measles cases and case definition send at different level of health system. Both public and private health facilities were involved. National reference laboratory was supplied with reagents and operational funds by the WHO and French embassy. Measles control measures were implemented to prevent disease spreading in schools and any international spread. Attendance to school was restricted for children affected by measles for about 2 weeks. Travellers´ surveillance was enhanced at point of entry surveillance to prevent international spread of measles cases to neighbouring island within the framework of international health regulation (IHR). All passengers entering Madagascar as well as those leaving were screened to find any sign of fever or measles rash.

**Strengthening routine immunization:** routine immunization strengthening workshop was organized at central level in order to identify problems, set up priorities and propose solutions. In addition, regions were prioritized and regions which will received the WHO support were identified. Finally, experts were recruited in order to provide support to routine immunization strengthening.

**Communication:** a communication team was set up and was in charge of developing a communication plan, posters, flyers and messages related to the outbreak, vaccination response and population sensitization. Communication team members attended coordination meetings.

**Logistics and supplies:** the country logistics and supply needs were expressed during coordination meetings. Partners chose logistics and supply needs to fulfill according to their field of intervention and worked in collaboration with the EPI at central level to make supplies available at district level.

## Results

**Epidemiological surveillance:** the outbreak was declared on October 4^th^, 2018. The previous outbreak occurred in Madagascar in 2003. The outbreak started in the main district of the capital, Antananarivo and spread to other districts. As of May 26^th^, 2019, 114 (100%) health districts were affected by the measles outbreak. From September 2018 to May 2019, a total of 146,277 measles cases were reported included 1394 (1%) laboratory-confirmed cases and 144,883 (99%) epidemiological link-confirmed cases. The outbreak affected equally males (72,917 cases; 49.85%) and females (73,233 cases; 50.06%). The sex was not specified for 127 (0.09%) cases. Measles cases age ranged from a minimum of 1 month to a maximum of 92 years. The median and the mean were 6 years and 9 years respectively. The most affected age group was 1-4 years with 29% of cases followed by 5-9 years with 20% of cases. Children aged 1 to 9 years accounted for 49% of measles cases. In addition, 77% of cases aged less than 15 years. Case fatality rate and attack rate were high among children less than 5 years. Highest attack rates were among children below one year and specifically those between 9-11 months (5,981 per 1,000,000 inhabitants) where attack rate was about 11 times the global attack rate (555 per 1,000,000 inhabitants).

**Figure 1:**
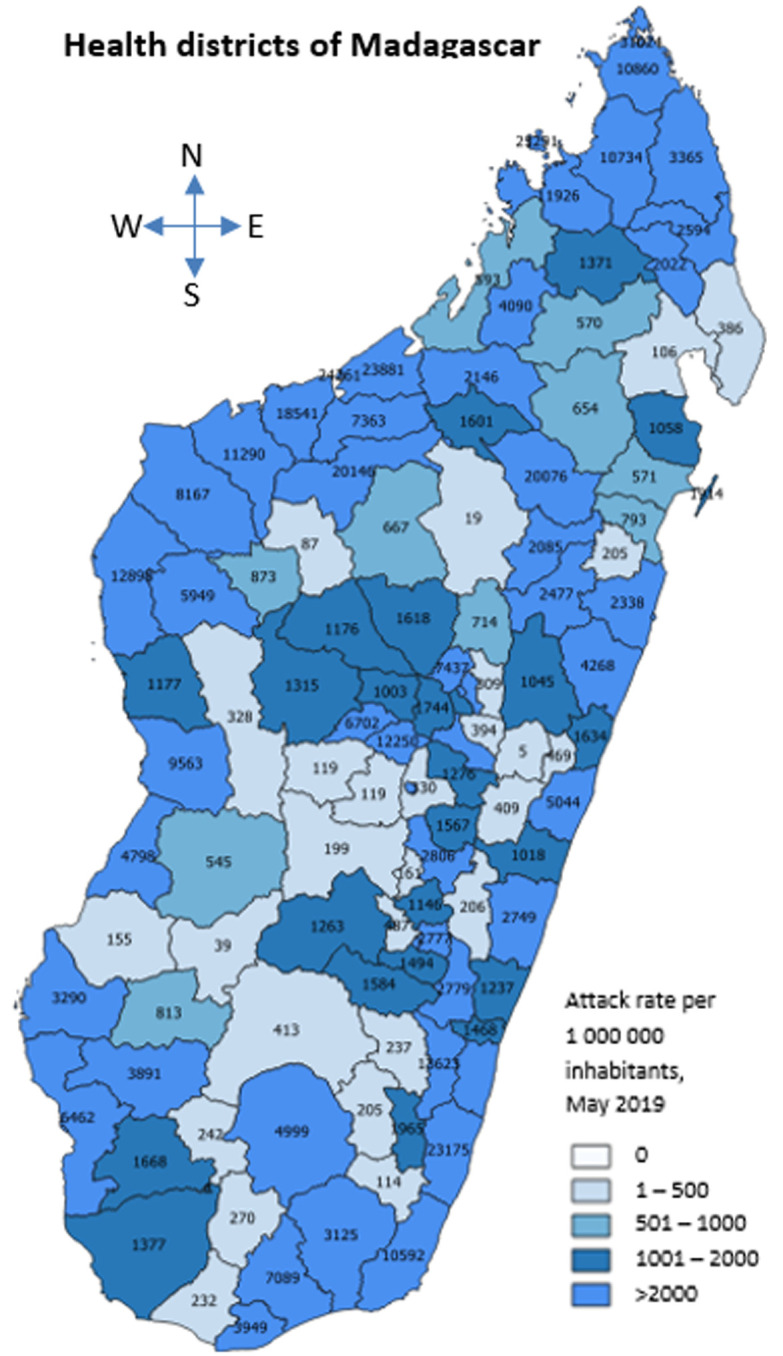
district specific attack rate, Madagascar, May 2019

A total of 97,713 (67%) cases were unvaccinated. During preliminary investigations, the five gingival samples collected and analysed in the national laboratory were all measles positive. Subsequently, genetic analysis performed by South Africa reference laboratory showed that wild measles virus B3 serotype was the causal agent of the outbreak. Overall, 71 (62.28%) districts had an attack rate greater than 2000 cases per 1,000,000 inhabitants, 22 (19.30%) districts had an attack rate of 1000-1999 cases per 1,000,000 inhabitants, 9 (7.90%) districts had an attack rate of 500-999 cases per 1,000,000 inhabitants, and 12 (10.52%) districts had an attack rate of 1-499 cases per 1,000,000 inhabitants (Figure 1). The daily notification of cases and deaths was strengthened from districts to central level where data base was regularly cleaned and available. Cases and deaths were investigated in four regions. In addition, rapid response teams were set up and members´ were trained in the south east and south west region. Overall, 8292 passengers and 583 crew members were screened at the points of entry.

**Case management:** heads of basic health facilities were briefed on case management protocol in 67 districts. Supportive supervision of care providers was organized in 39 districts. A total of 600 health workers were trained in 112 basic health facilities and 20 hospitals during formative supervision. Case management kits were provided to all 114 health districts to enable free of charge case management.

**Figure 2:**
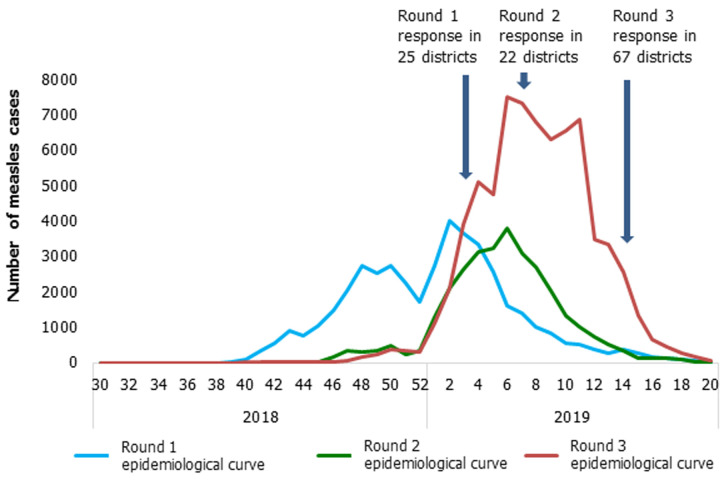
epidemiological curve evolution with the vaccination response, Madagascar, 3^rd^ September 2018 to 30^th^ May 2019

**Immunization response:** immunization response was implemented in three rounds. The first round took place from the 14^th^ to 18^th^ January 2019 in 25 districts and targeted children aged 9 months to 9 years. The second was implemented from 18 to 22 February 2019 in 22 districts and targeted children aged 6 months to 9 years. The third took place from the 25^th^ to 29^th^ March 2019 in 67 districts and targeted children aged 6 months to 9 years. A total of 7,265,990 children aged from 6 months to 9 years were vaccinated during the three round (101.9% administrative coverage). Independent monitoring showed an overall 4% of children missed during mass campaigns. The breakdown of this overall coverage showed an administrative coverage of 102%, 103.62% and 100.09% and 5%, 4% and 3% of non-vaccinated children during round 1, 2 and 3 respectively (Table 1). There was a decrease of the epidemiological curve after each vaccination response mainly in districts were vaccination campaign was implemented (Figure 2).

**Table 1:** reactive mass immunization campaigns findings during measles outbreak in Madagascar, January to March 2019

Round	Number of health districts targeted	Number of children targeted	Number vaccinated (nonzero dose)	Number vaccinated (zero dose)	Total vaccinated	Administrative coverage (%)	Number of AEFI notified	Proportion of children identified as non-vaccinated by independent monitoring (%)
Round 1	25	2,083,734	1,966,026	151,693	2,117,719	102.00	NA^*^	5
Round 2	22	1,160,767	1,030,420	172,364	1,202,784	103.62	33	4
Round 3	67	3,940,501	3571965	372,267	3,944,232	100.09	293	3
Total	114	7,185,002	6,568,411	696,324	7,264,735	101.90	326	4

^*^NA: not available; administrative coverage of each vaccination round was above the minimal target of 95% during measles outbreak in Madagascar, in 2019

**Routine immunization strengthening:** a workshop on the revitalization of the routine immunization was organized while full participation of the ministry of health (MoH), other ministries and partners. Major and pertinent interventions were identified and are being used to finalize the routine immunization strengthening plan. Financial resources are being mobilized to strengthen routine immunization. The Department for International Development (DFID) provided 1.3 million dollars to support the revitalization of the routine immunization. National authorities are fully committed to support routine immunization. A prioritization of regions to be supported for routine immunization was performed by WHO and human resources are being recruited.

**Figure 3:**
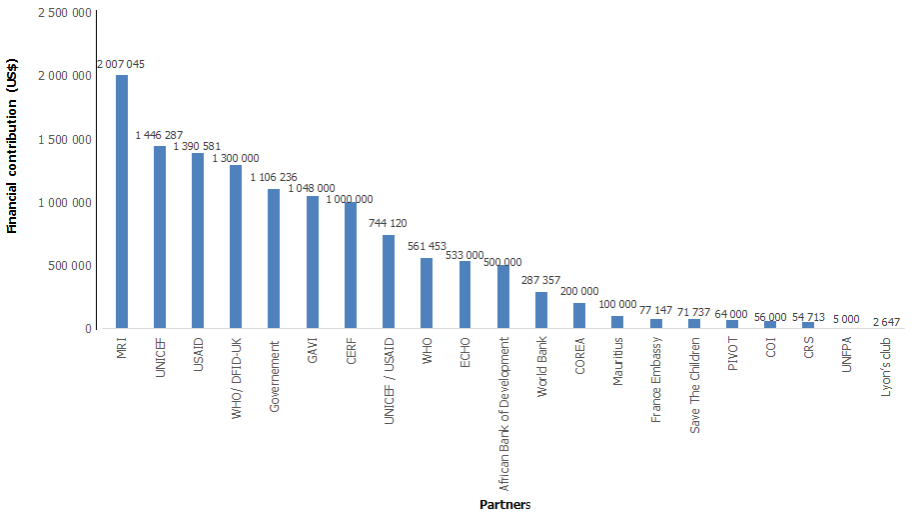
distribution of financial contribution by partners and government during measles outbreak, Madagascar, 3^rd^ September 2018 to 30^th^ May 2019

**Coordination and resources mobilisation:** strong collaborative work of all involved stakeholders under the lead of the MoH led to a coordinated mapping of partners and interventions, joint resources mobilization, efficient running on the emergency operation centre and regular national committee coordination meeting for decision making. This strengthened coordination led to an effective team spirit between the MoH and partners and enabled the effective implementation of response interventions. A total of US$ 12,555,323 were mobilized for the management of measles outbreak in Madagascar. This amount come from 21 partners and the government of Madagascar. Financial contribution ranged from a minimum of US$ 2,647 to a maximum of US$ 2,007,045. The four main contributors were MRI (16.00%), UNICEF (11.52%), USAID (11.08%) and WHO/DFID-UK (10.35%) (Figure 3).

**Communication:** under the overall lead of the MoH, communication was coordinated by UNICEF with involvement of WHO, USAID and partners. A total of 208 media partners were trained on the dissemination of quality information on measles and validated messages were disseminated on 208 partner media. Measles outbreak-related messages and vaccination campaign-related messages were disseminated in local languages in local radios. In addition, these messages were disseminated free of charge by telephone companies. A call center was activated. Of 21,293 calls received on the green line in March, 10,329 were related to measles. Thousands of fliers and posters were produced. WHO coordinated the risk communication interventions. Two press releases and 3 success stories were developed and disseminated. At least 10 interviews were conducted on international radio and television.

**Logistics:** WHO and UNICEF provided logisticians to strengthen the EPI capacities at central level. A plan for vaccine and consumables distribution and cases treatment kits distribution was developed. Logistician team at central level was in charge of reception and distribution of vaccine and consumables, vitamin A and treatment kits in health districts. A workshop was organized in order to strengthen the EPI staff capacity on logistics.

## Discussion

The current Madagascar measles outbreak occurred 15 years after the previous epidemic reported in 2003 [11,12]. The 2018 outbreak started in one health district of the capital city, Antananarivo and sprayed rapidly in the other health districts of the country. Six months after the beginning of the outbreak, 100% (114) of health districts were in epidemic. This extension of the outbreak across the entire country could be explained by many factors. Firstly, le late implementation of mass vaccination response. Indeed, vaccination response was implemented in 3 rounds due to the insufficient measles vaccines stockpiles globally and the limited financial resources (internal and external). Therefore, districts were prioritized on the basis of routine vaccination coverage, age-specific attack rates and absolute number of cases as recommended by the WHO [3,13]. The first round started 4 months after the beginning of the outbreak and included 22% (25) of health districts, the second round began a month after the first and targeted 19% (22) of health districts and the third round commenced a month after the second round and included 59% (67) of health districts [14-16]. Secondly, the poor vaccination coverage of routine immunization. From 2014 to 2018, measles vaccine administrative coverage was above 80%. However, the WHO-UNICEF estimates aver the same period showed that measles vaccine coverage was around 60% [11,12]. Thirdly, the high population mobility. Madagascar experience a high internal migration for growing social tension due to imbalances in opportunities and access to social services [17]. Fourthly, the high transmission rate of measles. With a basic reproductive number (the average number of secondary cases produced by a primary case in a completely susceptible population) of 12-18 [18], measles is one of the most contagious diseases of humans [19,20].

Despite the fact that mass immunization campaigns started 4 months after the beginning of the outbreak, these campaigns played an important role in stopping the epidemic. In fact, as we can see in Figure 2, there was a remarkable inflection of epidemiological curve after each mass immunization campaign especially in health districts where these campaigns took place. This inflection was prolonged by an evolution of the epidemiological curve toward abscissa axis, suggesting a reduction of the transmission and consequently a decrease of measles new cases which led to the end of the outbreak. Our findings support previous studies reporting that there is a long period between the occurrence of measles outbreaks and the implementation of vaccination response, and also the impact of vaccination campaigns on the spread of the disease [21-23]. Many factors contributed to the success of measles outbreak response in Madagascar. Firstly, a strong political will. There was a strong political will to put an end to the measles outbreak in Madagascar. This political will was characterized by an official launch of the vaccination campaign led by the president of the country accompanied by the minister of health and the WHO country representative. The political will favoured a massive participation of population in the vaccination campaigns. Secondly, a good coordination.

At central level, coordination meetings led by the health authorities were occasions to discuss age of target population for the vaccination response and vaccination period, to take decision for the official information of health care workers about the outbreak-related activities, to identify obstacle to the implementation of the outbreak management and propose solutions and to advocate to get support from partners. In addition, partners´ meetings leaded by the WHO were opportunities to discuss on the type and level of support partners could provide to Madagascar in accordance to the country´s needs. At subnational level, coordination activities were organised at regional and district level. These activities included activation of the outbreak management committees as in other countries [13]. Thirdly, the strengthening of epidemiological surveillance. Health worker capacity was built for measles clinical diagnosis. In addition, health facilities were provided with line-listing for cases recording and data transmission to central level. In accordance with the International Health Regulation (IHR), all travellers entering Madagascar as well as those leaving were screened to find any sign of fever or measles rash, to prevent international spread of measles cases. Fourthly, strengthening of communication activities. Communication activities were implemented as recommended by the WHO [24]. In fact, population awareness for measles vaccination campaigns was raised by the broadcast of vaccination-related information in national television and radio during pick hours. In addition, population was sensitized in churches and mosques and vaccination campaign-related posters were stuck in all public health facilities.

Moreover, press conferences were periodically organized by the ministry of health to keep population updated on the outbreak evolution and prevent the release of inaccurate information in media. Fifthly, a high vaccination coverage during mass campaigns. Each round of the outbreak vaccination response achieved the minimum coverage of 95% recommended by the WHO [3]. Sixth, partners financial support. A total of US$ 12,555,323 were mobilized for the management of measles outbreak in Madagascar. This financial support was of a huge importance since it covered the outbreak response-related expenses. Challenges faced during measles outbreak in Madagascar included low community-based surveillance, cases´ treatment kits shortage in health facilities due to inadequate supply, logistic problems and difficulty to access some districts and insufficiency of financial resources.

### Strengths and weaknesses of the study

**Strengths of our study:** data have been collected using a standardized tool; cases definition have been standardized; blood samples biological analysis has been performed by the national reference laboratory. Results of our study should be used taking into account its limitations. Firstly, financial resources for the outbreak response may be underestimated since resources mobilized after May 30^th^, 2019, were not included in our study. Secondly, the scope of the outbreak may have been underestimated since case included in our study were those recorded by health facilities. Cases and deaths which occurred in the community without visiting health facilities were not included in our study.

## Conclusion

Measles outbreak response in Madagascar required a strong political will backed by partners support. The strengthening of many areas was included in the outbreak response. Although the overall vaccination response coverage exceeded the minimum coverage target, there are some susceptible individuals in the country. Elimination of measles will be challenging in Madagascar because of low routine immunization coverage and the absence of a second dose of measles vaccine in the routine immunization schedule. Future studies could be conducted to understand reasons of low national immunization performances over the years and address the cost of the outbreak vaccination response.

### What is known about this topic

- Measles is one of the most contagious diseases of humans with a basic reproductive number (the average number of secondary cases produced by a primary case in a completely susceptible population) of 12-18;
- The risk of developing fatal or severe measles increases for children aged less than 5 years, living in overcrowded conditions, who are malnourished (especially with vitamin A deficiency);
- Failure to maintain high coverage of childhood immunization in all districts has resulted in a resurgence of the disease, in countries where vaccination has substantially reduced the incidence of measles.

### What this study adds

- The early mass immunization campaign against measles could have prevented many cases and the extension of the disease in all the health districts of the country;
- In a context of country wide outbreak with limited resources, the vaccination response can be implemented in different phases on the basis of districts prioritization;
- There is a need of having sufficient measles vaccine stockpiles at global level.
